# Distribution pattern of lymph node metastases and its implication in individualized radiotherapeutic clinical target volume delineation of regional lymph nodes in patients with stage IA to IIA cervical cancer

**DOI:** 10.1186/s13014-015-0352-5

**Published:** 2015-02-15

**Authors:** Xinglan Li, Yueju Yin, Xuigui Sheng, Xiaoyun Han, Li Sun, Chunhua Lu, Xiang Wang

**Affiliations:** Department of Gynecologic Oncology, Shandong Cancer Hospital and Institute, 440 Jiyan Road, Jinan, 250117 Shandong Province People’s Republic of China; School of Medicine and Life Sciences, University of Jinan - Shandong Academy of Medical Sciences, 106 Jiwei Road, Jinan, 250022 Shandong Province People’s Republic of China

**Keywords:** Cervical cancer, Pelvic lymphadenectomy, Radiotherapy, Target volume

## Abstract

**Background:**

To study the distribution pattern of lymph node metastases of stage IA to IIA cervical cancer and to clarify the individualized clinical target volume delineation of regional lymph nodes (CTVn).

**Methods:**

A total of 665 cases with International Federation Gynecology and Obstetrics stage IA to IIA cervical cancer who underwent radical hysterectomy and pelvic lymphadenectomy were retrospectively reviewed. The clinicopathological factors related to lymph node metastases were analyzed using logistic regression analysis.

**Results:**

Pelvic lymph node metastases were found in 168 of 665 patients resulting in a metastasis rate of 25.3%. Binary logistic regression analysis showed that age, lymph vascular space involvement, and deep stromal invasion statistically influenced pelvic lymph node metastases (p = 0.017, < 0.001, < 0.001, respectively). Pathological morphology type, lymph node metastases of the obturator, the external iliac and internal iliac, and the para-aortic had a strong influence on lymph node metastases of the common iliac (p = 0.022, 0.003, < 0.001, 0.009, respectively). Tumor size and lymph node metastases of the common iliac were significantly related to lymph node metastases of the para-aortic (p = 0.045, < 0.001, respectively). Lymph node metastases of the obturator, the external iliac and internal iliac were strongly correlated to lymph node metastases of the circumflex iliac node distal to the external iliac node (CINDEIN; p = 0.027, 0.024, respectively).

**Conclusions:**

Factors related to lymph node metastases should be comprehensively considered to design and tailor CTVn for radiotherapy of cervical cancer. Selective regional irradiation including the correlated lymphatic drainage regions should be performed.

## Background

Cervical cancer is the most common gynecological malignancy and the second most frequent cause of cancer death in Chinese women [1]. Radical hysterectomy and pelvic lymphadenectomy are the standard treatment for early stage cervical cancer. Postoperative radiotherapy is a standard component of multimodality treatments for patients with high-risk factors. Generally, patients with risk factors, such as positive pelvic nodes, parametrial invasion, or a positive vaginal margin are regarded as being at a “high risk” of recurrence [2].

Although the status of pelvic lymph nodes is not included in International Federation Gynecology and Obstetrics (FIGO) stage, it is one of the most important prognostic factors and also an indicator of the need for postoperative radiotherapy [3]. The study of risk factors related to lymph node metastases can help identify patients who are more likely to have involved lymph nodes, and to guide individualized radiotherapy. Taylor et al. mentioned that inclusion of all pelvic lymph nodes in the clinical target volume (CTV) may not be necessary for all patients [4]. Accurate target definition is vitally important to ensure that the target is not over- or under-treated, and the dose to surrounding normal tissues is limited. However, there are no detailed guidelines on the personalized delineation of radiotherapeutic clinical target volume for cervical cancer patients with radical hysterectomy and pelvic lymphadenectomy. In the present study, we analyzed the pattern of lymph node metastases in patients who had undergone radical hysterectomy and pelvic lymphadenectomy. The impact of lymph node metastasis on radiotherapeutic clinical target volume delineation of regional lymph nodes (CTVn) in cervical cancer was also investigated.

## Methods

We retrospectively studied 665 patients with 2009 FIGO [5] stage IA to IIA cervical cancer who had undergone radical hysterectomy and systematic lymphadenectomy at the Department of Gynecologic Oncology in Shandong Cancer Hospital between June 1999 and October 2013. The preoperative work-up included a complete history and physical examination. Computed tomography (CT)/X-ray (96.1%) of the chest and ultrasonography/CT (96.1%) of the abdomen, or positron emission tomography (PET)/CT (10.1%) were performed to exclude distant organ metastasis. Other essential conditions included Karnofsky Performance Scale (KPS) ≥ 70, with no preoperative chemotherapy and/or radiotherapy. Clinical and pathological data, including age, tumor size, FIGO stage, histological type, grade, deep stromal invasion, pathological morphology type, and lymph vascular space involvement (LVSI) were examined. Also included was the number of regional lymph nodes and the number of regional lymph nodes with metastases.

All patients underwent radical hysterectomy and bilateral pelvic lymphadenectomy by gynecologic oncologists. Para-aortic lymphadenectomy is not routinely performed in patients with cervical carcinoma during radical hysterectomy because the procedure increases both the morbidity and the operation time. The para-aortic lymphadenectomy was performed when there were positive pelvic nodes in the frozen section or when there were suspicious or enlarged pelvic or para-aortic lymph nodes, as determined by intraoperative palpation. Lymph tissues were completely removed along the common iliac, external iliac, and internal iliac vessels from above the common iliac bifurcation to the supra-inguinal ligament on both sides. In the present study, we grouped the dissected lymph nodes into six categories: the parametrial group is from vaginal cuff to medial edge of the internal obturator muscle/ischial ramus on each side; the obturator group is removed superior to the level of the obturator nerve; the external iliac and internal iliac group is located along the external and internal iliac arteries between the inguinal canal and below the bifurcation of the common iliac artery; the common iliac group is defined as the bifurcation level of the aorta to the bifurcation of common iliac arteries into external and internal iliac arteries; the circumflex iliac node distal to the external iliac node (CINDEIN) group is located between the inguinal ligament and the deep circumflex vein; the para-aortic group is from the level of the aortic bifurcation up to the level of renal vessels. Dissected lymphatic tissues were placed in different specimen bottles according to their origin and sent for pathological evaluation. The number of lymph nodes was evaluated and recorded by two pathologists.

## Statistics

The clinicopathological factors likely to influence lymph node metastasis in cervical cancer, including age, tumor size, FIGO stage, histological type, grade, deep stromal invasion, pathological morphology type, and LVSI were entered into statistical analysis. All parameters, such as odds ratio (OR) and 95% confidence interval (CI), were calculated with respect to their relationship with cervical cancer lymph node metastasis using forward step-wise binary logistic regression . All statistical tests were two-sided and p < 0.05 was considered statistically significant. All statistical analyses were performed using SPSS Statistics (Version 17.0; SPSS Inc., Chicago, IL, USA).

## Results

### Patients and clinicopathological features

A total of 665 patients were included in the present study. The median age was 44 years (range 22–77 years). In total, 3.3% of patients were FIGO stage IA1, 1.1% stage IA2, 22.2% stage IB1, 16.1% stage IB2, 29.9% stage IIA1, and 27.4% stage IIA2. There were 491 (73.8%) squamous cell carcinomas, 139 (20.9%) adenocarcinomas, and 35 (5.3%) other types of malignancies. Tumor diameter was less than 4 cm in 387 (58.2%) and larger than 4 cm in 278 (41.8%) patients. In terms of macroscopic types, there were 636 (95.6%) patients with exophytic type and 29 (4.4%) with endophytic type. Deep stromal invasion was present in 49.5% of the patients. The percentage of patients with positive LVSI was 10.8%. The clinical and pathological characteristics of all 665 patients are shown in Table 1.

**Table 1:** **Clinicopathological features**

**Characteristics**	**N = 665**
Age (years)	44 (22–77)
FIGO stage	
1 A1	22
1 A2	7
1 B1	148
1 B2	107
II A1	199
II A2	182
Histology	
Squamos cacinoma	587
Adenocarcinoma	587
Other type	32
Tumor size	
≤4 cm	387
>4 cm	278
Tumor grade	
G1	72
G2	355
G3	238
Pathologic morphology type	
Endophytic type	29
Exophytic	636
Deep stromal invasion	
≤1/2	329
>1/2	336
LVSI	
Positive	72
Negative	593

*Abbreviation*: *FIGO* International Federation Gynecology and Obstetrics, *LVSI* lymph vascular space involvement, *G* Grade.

### Overall pattern of lymph node metastases

**Figure 1:**
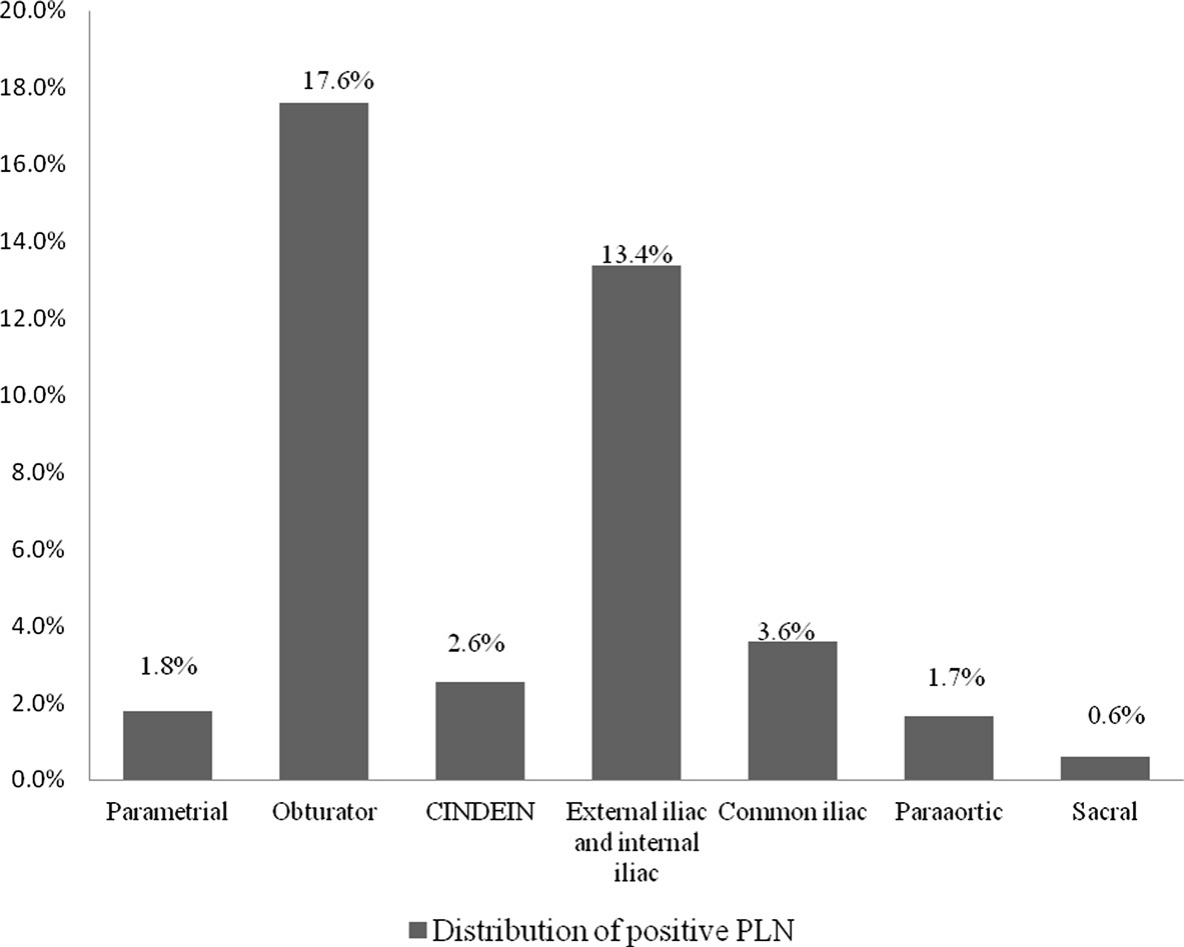
**Distribution of positive PLN in stage 1 A to 11 A cervical cancer.**

The median number of dissected lymph nodes was 19, with a range of 14–54. Pelvic lymph node metastases were found in 168 patients with a metastasis rate of 25.3%. The most common site for pelvic lymph node metastases was the obturator (17.6%; 117 of 665 patients), followed by the external iliac and internal iliac nodes (13.4%; 89 of 665 patients), common iliac nodes (3.6%; 24 of 665 patients), CINDEIN (2.6%; 17 of 665 patients), and parametrial nodes (1.8%; 12 of 665 patients). Metastases to the para-aortic (1.7%; 11 of 665 patients) and sacral nodes (0.6%; 4 of 665 patients) were relatively rare. The rates of lymph node metastasis in different groups of cervical cancer are shown in Figure 1.

### Factors associated with pelvic lymph node metastases

The relationships between pelvic lymph node metastasis rate and various clinical characteristics are shown in Table 2. We entered age, tumor size, macroscopic type, FIGO stage, histological type, grade, deep stromal invasion, pathological morphology type, and LVSI into the binary logistic statistical analysis. The results showed that age, deep stromal invasion, and LVSI had a statistical influence on pelvic lymph node metastases, while age decreased the risk of lymph node metastases (p = 0.017, OR = 0.975, 95% CI = 0.955–0.996). On the contrary, deep stromal invasion and LVSI increased the risk of lymph node metastases (p < 0.001, < 0.001, respectively; OR = 2.930, 3.967, respectively; 95% CI = 2.006–4.280, 2.358–6.674, respectively).

**Table 2:** **Multivariate analysis of risk factors associated with lymph node metastase**

	**B**	**S.E**	**Wald**	**df**	**p value**	**OR**	**95% CI for OR**
Age	−0.025	0.011	5.680	1	0.017	0.975	0.955-0.996
Deep stromal invasion	1.075	0.193	30.95	1	<0.001	2.930	2.006-4.280
LVSI	1.378	0.265	26.95	1	<0.001	3.967	2.358-6.674
Constant	−1.871	0.509	13.53	1	<0.001	0.154	

*Abbreviation*: *LVS*I lymph vascular space involvement, *OR* odds ratios, *CI* confidence interval.

### Factors associated with common iliac, para-aortic, and CINDEIN lymph node metastases

Table 3 shows the result of the multivariate analysis for factors associated with common iliac, para-aortic, and CINDEIN lymph node metastases. The binary logistic regression analysis showed that lymph node metastases of the common iliac was strongly influenced by pathological morphology type, lymph node metastases of the obturator, the external iliac and internal iliac, and the para-aortic (p = 0.022, 0.003, < 0.001, 0.009, respectively; OR = 5.572, 1.667, 1.858, 3.215, respectively; 95% CI = 1.285–24.16, 1.185–2.345, 1.345-2.566, 1.343–7.695, respectively). Tumor size and lymph node metastases of the common iliac were significantly related with lymph node metastases of the para-aortic (p = 0.045, < 0.001, respectively; OR = 5.165, 1.593, respectively; 95% CI = 1.036–25.76, 1.277–1.988, respectively). Lymph node metastases of the obturator, the external iliac and internal iliac were statistically correlated with CINDEIN lymph node metastases (p = 0.027, 0.024, respectively; OR = 1.419, 1.403, respectively; 95% CI = 1.041–1.934, 1.046–1.882, respectively).

**Table 3:** **Multivariate analysis of risk factors associated with common iliac, para-aortic lymph node and CINDEIN metastases in cervical cancer**

**Group**	**Parameters**	**B**	**S.E**	**Wals**	**df**	**p value**	**OR**	**95% CI for OR**
Common iliac	Pathologic morphology type	1.718	0.748	5.268	1	0.022	5.572	1.285-24.16
	LNM of obturator	0.511	0.174	8.630	1	0.003	1.667	1.185-2.345
	LNM of external iliac and internal iliac	0.619	0.165	14.10	1	<0.001	1.858	1.345-2.566
	LNM of para-aortic	1.168	0.445	6.881	1	0.009	3.215	1.343-7.695
Para-aortic	Tumor size	1.642	0.820	4.011	1	0.045	5.165	1.036-25.76
	LNM of common iliac	0.466	0.113	17.05	1	<0.001	1.593	1.277-1.988
CINDEIN	LNM of obturator	0.350	0.158	4.905	1	0.027	1.419	1.041-1.934
	LNM of external iliac and internal iliac	0.339	0.150	5.121	1	0.024	1.403	1.046-1.882

*Abbreviation*: *LNM* lymph node metastases, *CINDEIN* circumflexiliac node distal to the external iliac node, *OR* odds ratios, *CI* confidence interval.

## Discussion

Cervical cancer is clinically classified according to the FIGO clinical staging system, which does not include evaluation of lymph node status. However, lymphatic channel dissemination is one of common methods of cervical cancer spread. Lymph node status is the most important prognostic factor and has an immense impact on the subsequent determination of adjuvant therapy in early cervical carcinoma [6]. In a report by Peters et al. [7], recurrences are much more frequent in patients with lymph node involvement, and concurrent cisplatin-based chemotherapy (with or without 5-fluorouracil) together with pelvic irradiation is superior to radiation alone in improving progression-free and overall survival. Nevertheless, several studies have shown [7-12] that patients benefited from concurrent cisplatin with pelvic radiotherapy, but they also suffered from treatment toxicities. Acute grade 3–4 nonhematologic toxicity was present in 23.4% and chronic grade 4 toxicity was present in 5% of patients with postoperative chemo-radiation therapy [7,8]. Whitney et al. [9] pointed out that the late complication (grade 3–4) rate is up to 16.2% with cisplatin-based chemotherapy at 3 years in their study of stage IIB-IVA cervical cancer with negative para-aortic lymph nodes. These toxicity rates increase when chemotherapy and extended field radiotherapy are combined, and the rate of acute nonhematologic grade 3–4 toxicity was 81%, while chronic grade 3–4 toxicity was 40% with follow-up up to 38 months [10]. Despite the already high toxicity, the situation may worsen with time. Grade 3 toxicities at 3 and 5 years were 7.7% and 9.3%, but increased approximately 0.34% per year through 10–20 years in a retrospective analysis of 1,784 cervical cancer patients who were treated with radiation [11]. This underlines the need for improvements in radiotherapy delivery. One way to accomplish this is with the application of intensity-modulated radiotherapy (IMRT) particularly suitable for cervical cancer with irregular irradiation field. IMRT has been shown to reduce the incidence of acute and late toxicities and has been associated with low rates of in-field failures [13-15]. Mundt et al. [16,17] published results of patients treated with IMRT versus conventional radiotherapy. Grade 2 gastrointestinal symptoms were reduced from 91% to 60% and late gastrointestinal toxicity decreased from 50% to 11.1%. IMRT should be applied and combined with chemotherapy to reduce complications and improve the local control rates of cervical cancer patients. Another way to achieve this task is with accurate delineation of the target volume. Accurate target delineation is very important to avoid over-treatment. An over-treated target can increase the doses of normal tissues and result in more complications. However, at present, there are no consensus guidelines on the basis of different risk factors for the optimal delineation of radiotherapeutic CTVn of IMRT for cervical cancer patients with radical hysterectomy and lymphadenectomy. In the present study, we retrospectively examined 665 cervical cancer patients who had undergone radical hysterectomy and lymphadenectomy, and analyzed patterns of lymph node metastases. From these results, we intended to obtain useful information on how to define individualized CTVn for patients with cervical cancer who are slated to undergo IMRT.

In the present study, the most common site for pelvic lymph node metastases was the obturator, at a rate of 17.6%, followed by the external iliac and internal iliac (13.4%), common iliac (3.6%), and CINDEIN (2.6%). Metastases to parametrial (1.8%), para-aortic (1.7%) and presacral nodes (0.6%) were relatively rare. Our results confirmed the findings of Sakuragi et al. [18]. It has been shown that the depth of LVSI [18,19] and deep stromal invasion [18] are associated with lymphatic metastases in cervical cancer in previous studies. Similarly, our results suggested that independent prognostic factors for lymph node metastases include LVSI (p < 0.001) and deep stromal invasion (p < 0.001). Furthermore, we also found that age was a significant predictor of lymph node metastases (p = 0.017). The age range of patients in this study was 22 to 77 years old, and they were arbitrarily divided into two age groups, 40 years and under, and > 40 years. The percentage of positive nodes was higher in the younger group compared with the older group (≤ 40 years, 27.9% positive versus > 40 years, 23.7%). Our results showed that old age was a protective factor for lymph node metastases. However, this finding does not mean that older patients have lower risks of cervical cancer lymph node metastases, but our results emphasized the important role of age in cervical cancer lymph node metastases.

According to the distribution pattern of lymph node metastasis described above, personalized delineation of CTVn on the basis of different risk factors was considered. National Comprehensive Cancer Network (NCCN) Clinical Practice Guidelines (2013) [20] in cervical cancer provided us definitive guidelines for postoperative patients with negative nodes who would undergo radiotherapy. Using these guides, the radiation lymph region should at least include the parametria, presacral, obturator, internal iliac, and external iliac nodal regions whether or not lymph nodes are involved. Therefore, CTVn mainly focused on the common iliac, para-aortic, and CINDEIN in our present study. Although the NCCN (2013) [20] and the Radiation Therapy Oncology Group (RTOG; 2008) [21] achieved some consensus guidelines on a CTV definition for intensity-modulated pelvic radiotherapy for the postoperative treatment of cervical cancer, they mainly defined margins and rarely mentioned how to perform selective regional irradiation with different risk factors. The results of our subgroup analysis suggested that radiation oncologists should design individualized radiotherapeutic CTVns for cervical cancer patients with different risk factors.

Our subgroup analysis suggested that endophytic tumors were associated with an increased risk of common iliac lymph node metastasis. Our results emphasized the importance of pathological morphology type in cervical cancer lymph node metastases. Although common iliac node metastases are rare in early stage carcinomas in the absence of positive pelvic nodes [22], they should be included in CTVn for cervical cancer patients with endophytic tumors. Previous reports showed that positive lymph nodes are seldom found in the common iliac lymph area unless the pelvic nodes are involved, but they didn’t describe in detail which group involved would statistically increase risk [18,23]. Our study revealed that common iliac involvement was influenced by lymph node metastases of the obturator, the external iliac and internal iliac, and the para-aortic (p = 0.003, < 0.001, 0.009, respectively). We recommended delineating common iliac nodal region when these three above-mentioned groups are involved.

The survival outcome of cervical cancer patients with positive para-aortic lymph nodes is poor; a 30% 5-year survival has been demonstrated in a retrospective study of these patients [24]. Pelvic radiation therapy was required to improve survival rates with cure in 60% to 70% of Stage IIB [25], but there was a high incidence of gastrointestinal toxicity with conventional extended field radiotherapy and the dose is usually limited to 45 Gy because of normal tissue dose limitations. Portelance et al. [26] reported a 30% to 70% reduction in dose to organs at risk (OARs) with IMRT compared with a conventional four-field technique. Early data on the use of extended field IMRT and concurrent cisplatin chemotherapy was encouraging, with less late gastrointestinal morbidity than historical series at standard doses and using simultaneous integrated boost (SIB) IMRT for dose escalation to involved nodes [13,27,28]. Although IMRT can increase the dose to the target and reduce the dose in normal tissues, accurate delineation is very important for IMRT to ensure that the target is not under- or over-treatment. IMRT together with accurate delineation can further decrease complications yet retain efficacy. We found that tumor size and lymph node metastasis of the common iliac were associated with lymph node metastasis of the para-aortic. Our results were partially consistent with a previously published study [23] that showed that tumor size was a prognostic factor of lymph node metastasis of the para-aortic (p < 0.01). Presence of pelvic lymph node metastasis increases the risk of para-aortic involvement in several studies [18,22,23,29]. In our study, we found that common iliac involvement predicted para-aortic lymph node metastasis. When tumor size was > 4 cm or lymph node metastasis of the common iliac is indicated, we recommend including the para-aortic lymph nodes in the treatment volume.

CINDEIN, which is the most distal external iliac lymph node in the pelvic cavity, has been called the lateral deep inguinal node [18], suprainguinal node [29], circumflex iliac node [30], and distal external iliac lymph node [31] in the literature. This group of lymph nodes is usually included in CTV in previous studies [32,33], but seldom mentioned with specific contouring recommendations. Several reports, including our previous study [29,31,34], have described the incidence of the CINDEIN metastasis in cervical cancer from 2.5% to 8% and have demonstrated that CINDEIN is seldom involved in metastasis. In the present study, we found that 17 of 665 patients had CINDEIN involvement and the incidence of CINDEIN metastases was 2.6%. Of these 17 patients, 16 had obturator or internal and external iliac involvement, while only one patient without other groups of lymph node metastases had positive CINDEIN. Patients without CINDEIN involvement was 97.4% (648 of 665). Of these 648 patients, 146 had obturator or internal and external iliac involvement, 2 had isolated parametrial node involvement, 2 had isolated common iliac involvement, and 498 had negative lymph node. The negative predictive value of the negative other groups on CINDEIN metastasis in patients with early stage cervical cancer was therefore 99.8% (498 of 499 patients). Together with our subgroup analysis, we definitively showed that the obturator involvement, and the internal and external iliac involvement were risk factors for CINDEIN metastasis. Thus, CINDEIN metastases might occur subsequently to widespread pelvic lymph node metastasis, especially in the obturator, and the external and internal iliac regions. This is consistent with the routes of lymph flow from the uterine cervix, as described in previous reports [35,36]. Therefore, CINDEIN is recommended to be contoured into CTV when the obturator or the internal and external iliac become involved. Otherwise, it is not suggested in CTV with negative obturator, internal and external iliac lymph nodes, and especially with no lymph node involvement.

## Conclusion

CTVn must be customized by experienced oncologists according to the various clinical factors that influence lymph node metastasis. Irradiation of selective regional lymph nodes and their correlated lymphatic drainage regions should be performed according to clinical and pathological factors, deep stromal invasion, and LVSI. For large and endophytic tumors, the irradiation field should be enlarged appropriately. Our results can improve the accuracy of postoperative adjuvant therapy and allow a more individualized treatment for cervical cancer patients.
